# 2-Phenyl-3-(trimethyl­sil­yl)propan-1-aminium chloride

**DOI:** 10.1107/S1600536811035410

**Published:** 2011-09-03

**Authors:** Yousef M. Hijji, Ray J. Butcher, Jerry P. Jasinski, Zachary White, Robert C. Rosenberg

**Affiliations:** a Chemistry Department, Morgan State University, 1700 East Cold Spring Lane, Baltimore, MD 21251, USA; b Department of Chemistry, Howard University, 525 College Street NW, Washington, DC 20059, USA; c Department of Chemistry, Keene State College, 229 Main Street, Keene, NH 03435-2001, USA

## Abstract

The title compound, C_12_H_22_NSi^+^·Cl^−^, contains two formula units in the asymmetric unit and is a hydro­chloride salt in which the amine N atom is protonated and the NH_3_^+^ group forms hydrogen bonds with the Cl^−^ anion, forming a ribbon in the *c*-axis direction.

## Related literature

For silicon-substituted β-phenyl­ethyl amine and its biological activity, see: Frankel *et al.* (1968 ▶). For applications of β-phenyl­ethyl amine in alkaloid synthesis *via* the Pictet–Spengler reaction, see: Lorenz *et al.* (2010 ▶). For uses and applications of 3-amino-propyl­silanes in nano technology and self-assembled monolayers, see: Li *et al.* (2009 ▶) and in reverse ionic liquids in oil extraction, see: Blasucci *et al.* (2010 ▶). For a description of the Cambridge Structural Database, see: Allen (2002 ▶).
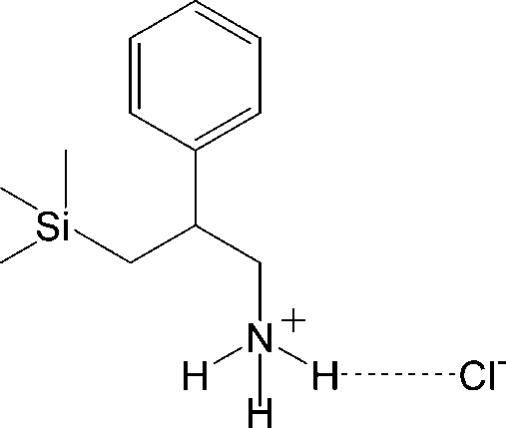


## Experimental

### 

#### Crystal data

C_12_H_22_NSi^+^·Cl^−^
*M* *_r_* = 243.85
Monoclinic, 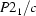

*a* = 12.3716 (4) Å
*b* = 32.6920 (8) Å
*c* = 7.44256 (18) Å
β = 93.006 (2)°
*V* = 3006.01 (14) Å^3^
*Z* = 8
Cu *K*α radiation
μ = 2.79 mm^−1^
*T* = 295 K
0.47 × 0.10 × 0.06 mm

#### Data collection

Oxford Diffraction Xcalibur Ruby Gemini diffractometer
Absorption correction: multi-scan (*CrysAlis PRO*; Oxford Diffraction, 2009 ▶) *T*_min_ = 0.370, *T*_max_ = 1.000
11195 measured reflections
5882 independent reflections
3078 reflections with *I* > 2σ(*I*)
*R*_int_ = 0.049

#### Refinement

*R*[*F* ^2^ > 2σ(*F* ^2^)] = 0.076
*wR*(*F* ^2^) = 0.276
*S* = 1.14
5882 reflections
279 parameters
H-atom parameters constrained
Δρ_max_ = 0.72 e Å^−3^
Δρ_min_ = −0.49 e Å^−3^

### 

Data collection: *CrysAlis PRO* (Oxford Diffraction, 2009 ▶); cell refinement: *CrysAlis PRO*; data reduction: *CrysAlis PRO*; program(s) used to solve structure: *SHELXS97* (Sheldrick, 2008 ▶); program(s) used to refine structure: *SHELXL97* (Sheldrick, 2008 ▶); molecular graphics: *SHELXTL* (Sheldrick, 2008 ▶); software used to prepare material for publication: *SHELXTL*.

## Supplementary Material

Crystal structure: contains datablock(s) I, global. DOI: 10.1107/S1600536811035410/hg5087sup1.cif

Structure factors: contains datablock(s) I. DOI: 10.1107/S1600536811035410/hg5087Isup2.hkl

Supplementary material file. DOI: 10.1107/S1600536811035410/hg5087Isup3.cml

Additional supplementary materials:  crystallographic information; 3D view; checkCIF report

## Figures and Tables

**Table 1:** Hydrogen-bond geometry (Å, °)

*D*—H⋯*A*	*D*—H	H⋯*A*	*D*⋯*A*	*D*—H⋯*A*
N1*A*—H1*AA*⋯Cl2^i^	0.89	2.23	3.114 (5)	173
N1*A*—H1*AB*⋯Cl1	0.89	2.25	3.136 (4)	172
N1*A*—H1*AC*⋯Cl1^ii^	0.89	2.36	3.168 (5)	152
N1*B*—H1*BA*⋯Cl2^ii^	0.89	2.30	3.166 (4)	163
N1*B*—H1*BB*⋯Cl2	0.89	2.28	3.165 (4)	171
N1*B*—H1*BC*⋯Cl1	0.89	2.35	3.222 (5)	165

Symmetry codes: (i) 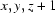; (ii) 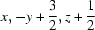.

**Fig. 1.**
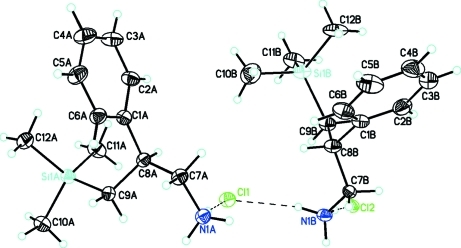
Diagram of C12H22ClNSi, showing the contents of the asymmetric unit. Hydrogen bonds are shown by dashed lines (30% atomic displacement parameters).

**Fig. 2.**
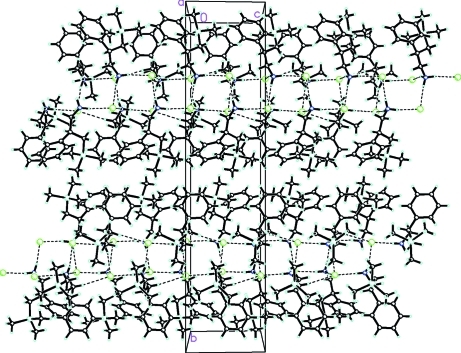
The molecular packing for C12H22ClNSi, viewed down the a axis showing the hydogen bonded ribbons in the c direction. Hydrogen bonds are shown by dashed lines.

## supplementary crystallographic information

### Comment

The title compound is a substituted α-phenyethylaminium chloride. Phenylethyl amines are substrates for dopamine-β-hydroxylase and are of biological importance. Silicon substituted phenylethyl amines have been investigated for biological activity and use as insecticide and applications in pharmaceuticals (Frankel *et al.* 1968). Viewing these compounds as substituted 3-silylpropylamine where they have application in monolayer construction and nanotechnology (Li *et al.* 2009) and use in oil recovery *via* reverse ionic liquids (Blasucci *et al.*, 2010). Phenylethyl amines are important building blocks in isoquinoline alkaloid synthesis *via* Pictet–Spengler (Lorenz *et al.* 2010).

In view of the importance of these compounds the structure of 2-phenyl-3-(trimethylsilyl)-propan-l-aminium chloride, C_12_H_22_ClNSi is reported. The title compound contains two formula units in the asymmetric unit and is a hydrochloride salt where the amine N is protonated and the NH_3_^+^ group forms hydrogen bonds with the Cl^-^ anion. These hydrogen bonds form a ribbon in the c direction. The metrical parameters for the salt are in the normal range (Allen, 2002).

### Experimental

To 5.20 g (44.4 mmol) benzylnitrile in 40 ml of dry THF under nitrogen atmosphere, cooled in an ice bath was added 28.0 ml of n-Bu Li (1.6 *M*) (44.8 mmol) dropwise. After the addition was complete the solution turned to a creamy slurry. The mixture was stirred for 10 minutes then the ice bath was removed and 5.54 g of chloromethyltrimethyl silane (6.3 ml) was added dropwise. After the addition was complete the mixture was stirred for 2 h at room temperature. The reaction was worked up by water addition and extraction with ether twice (25 ml). The organic layers were combined and washed with saturated NaCl solution, dried (MgSO4). The solvent was removed to give 3-trimethylsilyl-2-phenyl-propionitrile, as yellowish liquid 7.5 g (78%). 2.0 g of 3-trimethylsilyl-2-phenyl-propionitrile were dissolved in 5 ml of dry THF and heated to 343 K in a distillation set up. 3.0 ml of BH_3_.DMS (10 *M*) was added dropwise over a period of 10 minutes. Dimethylsulfide (DMS) distilled off the reaction mixture and was collected in the receiver. The mixture was heated for 15 minutes then cooled to room temperature. A reflux condenser was connected to the reaction flask and 10 ml of 6 *M* HCl was added carefully and slowly. After the addition was complete and no more gas evolved the mixture was heated for 30 minutes at reflux. The reaction mixture was cooled to room temperature, transferred to a beaker. KOH pellets were added slowly to the solution to neutralize the acid. The mixture was extracted with 2x25 ml of ether. The organic layers were combined and 5 ml of concentrated HCl was added. The aqueous layer was allowed to evaporate to give white solid. The solid was washed with ether and filtered to give 0.98 g (41%) of the title compound. A sample was dissolved in water and allowed to evaporate slowly to give clear crystals of the title compound used for *x*-ray crystallography.

^1^H NMR (DMSO-d_6_, 400 MHz): δ (p.p.m.) = 7.85 (br, 3H), 7.31 (m, 5H), 2.94 (m,3*H*), 1.00 (dd,1*H*, J= 14.5, 3.5 Hz), 0.92 (dd, 1 H, J = 14.5, 11 Hz), -0.28 (s, 9H). ^13^C NMR (DMSO-d_6_,, 100 MHz): δ (p.p.m.) 142.24, 128.65, 127.91, 127.16, 46.89, 39.78, 21.01, -1.21. Exact Mass = 207.084401 (*M*^+^ - HCl) Mass Spec (EI) direct probe *M*/*z*: 208 (M—Cl), 192 (*M*+ –NH_2_Cl), 177 (M—CH_2_NH_3_Cl), 147, 121, 104, 91, 73 (base).

### Refinement

H atoms were placed in geometrically idealized positions and constrained to ride on their parent atoms with a C—H distances of 0.93 to 0.97 Å and N—H distances of 0.89 Å and *U*_iso_(H) = 1.2*U*_eq_(C, N).

### Crystal data

**Table:**

C_12_H_22_NSi^+^·Cl^−^	*F*(000) = 1056
*M**_r_* = 243.85	*D*_x_ = 1.078 Mg m^−^^3^
Monoclinic, *P*2_1_/*c*	Cu *K*α radiation, λ = 1.54184 Å
Hall symbol: -P 2ybc	Cell parameters from 3131 reflections
*a* = 12.3716 (4) Å	θ = 4.5–75.7°
*b* = 32.6920 (8) Å	µ = 2.79 mm^−^^1^
*c* = 7.44256 (18) Å	*T* = 295 K
β = 93.006 (2)°	Needle, colorless
*V* = 3006.01 (14) Å^3^	0.47 × 0.10 × 0.06 mm
*Z* = 8	

### Data collection

**Table:**

Oxford Diffraction Xcalibur Ruby Gemini diffractometer	5882 independent reflections
Radiation source: Enhance (Cu) X-ray Source	3078 reflections with *I* > 2σ(*I*)
graphite	*R*_int_ = 0.049
Detector resolution: 10.5081 pixels mm^-1^	θ_max_ = 76.0°, θ_min_ = 4.5°
ω scans	*h* = −15→15
Absorption correction: multi-scan (*CrysAlis PRO*; Oxford Diffraction, 2009)	*k* = −40→38
*T*_min_ = 0.370, *T*_max_ = 1.000	*l* = −9→8
11195 measured reflections	

### Refinement

**Table:**

Refinement on *F*^2^	Primary atom site location: structure-invariant direct methods
Least-squares matrix: full	Secondary atom site location: difference Fourier map
*R*[*F*^2^ > 2σ(*F*^2^)] = 0.076	Hydrogen site location: inferred from neighbouring sites
*wR*(*F*^2^) = 0.276	H-atom parameters constrained
*S* = 1.14	*w* = 1/[σ^2^(*F*_o_^2^) + (0.1129*P*)^2^ + 1.1585*P*] where *P* = (*F*_o_^2^ + 2*F*_c_^2^)/3
5882 reflections	(Δ/σ)_max_ = 0.001
279 parameters	Δρ_max_ = 0.72 e Å^−^^3^
0 restraints	Δρ_min_ = −0.49 e Å^−^^3^

### Special details

**Table:**

Geometry. All e.s.d.'s (except the e.s.d. in the dihedral angle between two l.s. planes) are estimated using the full covariance matrix. The cell e.s.d.'s are taken into account individually in the estimation of e.s.d.'s in distances, angles and torsion angles; correlations between e.s.d.'s in cell parameters are only used when they are defined by crystal symmetry. An approximate (isotropic) treatment of cell e.s.d.'s is used for estimating e.s.d.'s involving l.s. planes.
Refinement. Refinement of *F*^2^ against ALL reflections. The weighted *R*-factor *wR* and goodness of fit *S* are based on *F*^2^, conventional *R*-factors *R* are based on *F*, with *F* set to zero for negative *F*^2^. The threshold expression of *F*^2^ > σ(*F*^2^) is used only for calculating *R*-factors(gt) *etc*. and is not relevant to the choice of reflections for refinement. *R*-factors based on *F*^2^ are statistically about twice as large as those based on *F*, and *R*- factors based on ALL data will be even larger.

### Fractional atomic coordinates and isotropic or equivalent isotropic displacement parameters (Å^2^)

**Table:**

	*x*	*y*	*z*	*U*_iso_*/*U*_eq_	
Cl1	0.51262 (14)	0.70004 (5)	0.46785 (17)	0.0747 (4)	
Cl2	0.24050 (12)	0.70213 (4)	−0.04653 (17)	0.0658 (4)	
Si1A	0.87067 (14)	0.68876 (5)	0.88371 (19)	0.0642 (4)	
N1A	0.4863 (4)	0.70536 (13)	0.8840 (5)	0.0643 (11)	
H1AA	0.4150	0.7050	0.8943	0.096*	
H1AB	0.5008	0.7041	0.7682	0.096*	
H1AC	0.5135	0.7284	0.9314	0.096*	
C1A	0.6903 (5)	0.62195 (16)	1.0207 (6)	0.0592 (13)	
C2A	0.7098 (6)	0.58664 (18)	0.9246 (8)	0.0757 (17)	
H2AA	0.6982	0.5864	0.8001	0.091*	
C3A	0.7464 (7)	0.5518 (2)	1.0127 (10)	0.099 (2)	
H3AA	0.7578	0.5282	0.9467	0.118*	
C4A	0.7661 (7)	0.5512 (2)	1.1946 (10)	0.103 (2)	
H4AA	0.7931	0.5278	1.2518	0.123*	
C5A	0.7459 (6)	0.5855 (2)	1.2916 (8)	0.089 (2)	
H5AA	0.7583	0.5853	1.4159	0.107*	
C6A	0.7070 (5)	0.62050 (18)	1.2066 (7)	0.0709 (15)	
H6AA	0.6918	0.6434	1.2749	0.085*	
C7A	0.5364 (5)	0.66953 (18)	0.9814 (7)	0.0670 (15)	
H7AA	0.4916	0.6456	0.9576	0.080*	
H7AB	0.5381	0.6748	1.1098	0.080*	
C8A	0.6509 (4)	0.66056 (15)	0.9266 (6)	0.0553 (12)	
H8AA	0.6463	0.6547	0.7972	0.066*	
C9A	0.7297 (4)	0.69618 (16)	0.9563 (7)	0.0582 (12)	
H9AA	0.7338	0.7027	1.0837	0.070*	
H9AB	0.6992	0.7198	0.8934	0.070*	
C10A	0.9367 (6)	0.73979 (18)	0.8718 (7)	0.0756 (16)	
H10A	0.9543	0.7496	0.9914	0.113*	
H10B	0.8882	0.7587	0.8103	0.113*	
H10C	1.0017	0.7374	0.8076	0.113*	
C11A	0.8624 (6)	0.6646 (2)	0.6552 (9)	0.092 (2)	
H11A	0.8152	0.6805	0.5760	0.138*	
H11B	0.8346	0.6373	0.6638	0.138*	
H11C	0.9333	0.6637	0.6087	0.138*	
C12A	0.9525 (6)	0.6562 (2)	1.0466 (9)	0.093 (2)	
H12A	0.9467	0.6667	1.1662	0.140*	
H12B	1.0270	0.6566	1.0162	0.140*	
H12C	0.9259	0.6286	1.0409	0.140*	
Si1B	0.36327 (18)	0.56463 (5)	0.3612 (3)	0.0814 (5)	
N1B	0.2550 (4)	0.70271 (12)	0.3793 (6)	0.0649 (12)	
H1BA	0.2379	0.7281	0.4103	0.097*	
H1BB	0.2464	0.7000	0.2604	0.097*	
H1BC	0.3236	0.6976	0.4142	0.097*	
C1B	0.1599 (6)	0.60320 (17)	0.5886 (8)	0.0731 (16)	
C2B	0.0698 (7)	0.5861 (2)	0.4979 (11)	0.097 (2)	
H2BA	0.0530	0.5928	0.3783	0.116*	
C3B	0.0038 (7)	0.5584 (2)	0.5886 (14)	0.113 (3)	
H3BA	−0.0559	0.5466	0.5276	0.136*	
C4B	0.0263 (9)	0.5491 (3)	0.7602 (14)	0.115 (3)	
H4BA	−0.0173	0.5306	0.8181	0.137*	
C5B	0.1124 (10)	0.5663 (3)	0.8517 (11)	0.118 (3)	
H5BA	0.1265	0.5599	0.9724	0.142*	
C6B	0.1802 (7)	0.5935 (2)	0.7679 (9)	0.092 (2)	
H6BA	0.2388	0.6052	0.8323	0.110*	
C7B	0.1835 (4)	0.67343 (15)	0.4673 (7)	0.0575 (12)	
H7BA	0.1168	0.6703	0.3940	0.069*	
H7BB	0.1652	0.6843	0.5831	0.069*	
C8B	0.2369 (6)	0.63141 (17)	0.4947 (7)	0.0717 (16)	
H8BA	0.2988	0.6357	0.5804	0.086*	
C9B	0.2827 (6)	0.61330 (18)	0.3298 (8)	0.0767 (17)	
H9BA	0.3288	0.6337	0.2778	0.092*	
H9BB	0.2232	0.6080	0.2428	0.092*	
C10B	0.4703 (9)	0.5713 (3)	0.5428 (13)	0.140 (4)	
H10D	0.5253	0.5510	0.5308	0.210*	
H10E	0.4392	0.5685	0.6576	0.210*	
H10F	0.5017	0.5981	0.5339	0.210*	
C11B	0.4268 (8)	0.5546 (2)	0.1443 (11)	0.115 (3)	
H11D	0.4694	0.5300	0.1546	0.173*	
H11E	0.4725	0.5771	0.1162	0.173*	
H11F	0.3714	0.5513	0.0503	0.173*	
C12B	0.2769 (8)	0.52048 (19)	0.4136 (12)	0.120 (3)	
H12D	0.3190	0.4958	0.4123	0.181*	
H12E	0.2177	0.5186	0.3252	0.181*	
H12F	0.2491	0.5241	0.5306	0.181*	

### Atomic displacement parameters (Å^2^)

**Table:**

	*U*^11^	*U*^22^	*U*^33^	*U*^12^	*U*^13^	*U*^23^
Cl1	0.0932 (11)	0.0737 (9)	0.0567 (7)	0.0071 (8)	−0.0001 (6)	−0.0029 (6)
Cl2	0.0764 (9)	0.0589 (7)	0.0617 (7)	−0.0045 (7)	−0.0010 (6)	0.0030 (5)
Si1A	0.0727 (10)	0.0614 (9)	0.0584 (8)	−0.0013 (8)	0.0027 (7)	−0.0008 (6)
N1A	0.069 (3)	0.068 (3)	0.056 (2)	0.006 (2)	0.000 (2)	−0.001 (2)
C1A	0.071 (3)	0.058 (3)	0.050 (2)	−0.004 (3)	0.003 (2)	0.003 (2)
C2A	0.105 (5)	0.058 (3)	0.065 (3)	−0.002 (3)	0.006 (3)	−0.006 (3)
C3A	0.135 (7)	0.055 (4)	0.107 (5)	0.009 (4)	0.018 (5)	−0.006 (3)
C4A	0.140 (8)	0.072 (4)	0.097 (5)	0.015 (5)	0.008 (5)	0.026 (4)
C5A	0.108 (6)	0.092 (5)	0.067 (3)	0.021 (4)	−0.003 (3)	0.021 (3)
C6A	0.085 (4)	0.067 (3)	0.060 (3)	0.009 (3)	−0.004 (3)	0.002 (3)
C7A	0.078 (4)	0.072 (4)	0.051 (2)	0.009 (3)	0.002 (2)	0.007 (2)
C8A	0.060 (3)	0.060 (3)	0.046 (2)	0.007 (2)	0.001 (2)	−0.002 (2)
C9A	0.057 (3)	0.059 (3)	0.058 (3)	0.002 (2)	−0.004 (2)	0.000 (2)
C10A	0.092 (5)	0.071 (4)	0.064 (3)	−0.010 (3)	0.010 (3)	0.001 (3)
C11A	0.102 (5)	0.092 (5)	0.086 (4)	−0.011 (4)	0.033 (4)	−0.028 (4)
C12A	0.095 (5)	0.077 (4)	0.105 (5)	0.007 (4)	−0.017 (4)	0.018 (4)
Si1B	0.1019 (14)	0.0555 (9)	0.0882 (11)	0.0095 (10)	0.0187 (10)	0.0047 (8)
N1B	0.086 (3)	0.048 (2)	0.060 (2)	0.001 (2)	−0.010 (2)	0.0017 (18)
C1B	0.090 (5)	0.048 (3)	0.082 (4)	0.003 (3)	0.006 (3)	−0.001 (3)
C2B	0.101 (6)	0.081 (5)	0.108 (5)	0.017 (4)	0.003 (4)	0.017 (4)
C3B	0.097 (6)	0.079 (5)	0.162 (8)	0.000 (5)	−0.003 (6)	0.013 (5)
C4B	0.128 (8)	0.080 (5)	0.140 (8)	−0.004 (5)	0.043 (6)	0.017 (5)
C5B	0.180 (10)	0.089 (6)	0.091 (5)	−0.012 (6)	0.048 (6)	0.005 (4)
C6B	0.128 (6)	0.073 (4)	0.076 (4)	−0.012 (4)	0.018 (4)	−0.002 (3)
C7B	0.061 (3)	0.049 (3)	0.062 (3)	−0.005 (2)	−0.004 (2)	−0.001 (2)
C8B	0.095 (5)	0.053 (3)	0.067 (3)	0.002 (3)	0.006 (3)	0.006 (2)
C9B	0.096 (5)	0.060 (3)	0.074 (3)	0.000 (3)	0.005 (3)	0.005 (3)
C10B	0.148 (9)	0.140 (9)	0.128 (7)	0.027 (7)	−0.026 (7)	−0.006 (6)
C11B	0.154 (8)	0.082 (5)	0.114 (6)	−0.010 (5)	0.039 (6)	−0.005 (4)
C12B	0.172 (9)	0.048 (4)	0.148 (7)	0.012 (5)	0.070 (6)	0.012 (4)

### Geometric parameters (Å, °)

**Table:**

Si1A—C10A	1.862 (6)	Si1B—C12B	1.850 (8)
Si1A—C9A	1.868 (6)	Si1B—C10B	1.855 (9)
Si1A—C12A	1.870 (6)	Si1B—C11B	1.861 (8)
Si1A—C11A	1.872 (6)	Si1B—C9B	1.886 (6)
N1A—C7A	1.495 (6)	N1B—C7B	1.479 (7)
N1A—H1AA	0.8900	N1B—H1BA	0.8900
N1A—H1AB	0.8900	N1B—H1BB	0.8900
N1A—H1AC	0.8900	N1B—H1BC	0.8900
C1A—C2A	1.386 (7)	C1B—C6B	1.381 (9)
C1A—C6A	1.389 (7)	C1B—C2B	1.391 (10)
C1A—C8A	1.512 (7)	C1B—C8B	1.522 (8)
C2A—C3A	1.380 (9)	C2B—C3B	1.414 (11)
C2A—H2AA	0.9300	C2B—H2BA	0.9300
C3A—C4A	1.363 (10)	C3B—C4B	1.328 (12)
C3A—H3AA	0.9300	C3B—H3BA	0.9300
C4A—C5A	1.364 (10)	C4B—C5B	1.357 (13)
C4A—H4AA	0.9300	C4B—H4BA	0.9300
C5A—C6A	1.381 (8)	C5B—C6B	1.393 (11)
C5A—H5AA	0.9300	C5B—H5BA	0.9300
C6A—H6AA	0.9300	C6B—H6BA	0.9300
C7A—C8A	1.523 (8)	C7B—C8B	1.534 (7)
C7A—H7AA	0.9700	C7B—H7BA	0.9700
C7A—H7AB	0.9700	C7B—H7BB	0.9700
C8A—C9A	1.527 (7)	C8B—C9B	1.500 (8)
C8A—H8AA	0.9800	C8B—H8BA	0.9800
C9A—H9AA	0.9700	C9B—H9BA	0.9700
C9A—H9AB	0.9700	C9B—H9BB	0.9700
C10A—H10A	0.9600	C10B—H10D	0.9600
C10A—H10B	0.9600	C10B—H10E	0.9600
C10A—H10C	0.9600	C10B—H10F	0.9600
C11A—H11A	0.9600	C11B—H11D	0.9600
C11A—H11B	0.9600	C11B—H11E	0.9600
C11A—H11C	0.9600	C11B—H11F	0.9600
C12A—H12A	0.9600	C12B—H12D	0.9600
C12A—H12B	0.9600	C12B—H12E	0.9600
C12A—H12C	0.9600	C12B—H12F	0.9600
			
C10A—Si1A—C9A	108.4 (3)	C12B—Si1B—C10B	109.7 (5)
C10A—Si1A—C12A	108.5 (3)	C12B—Si1B—C11B	108.7 (4)
C9A—Si1A—C12A	111.6 (3)	C10B—Si1B—C11B	109.6 (5)
C10A—Si1A—C11A	109.7 (3)	C12B—Si1B—C9B	112.1 (4)
C9A—Si1A—C11A	108.1 (3)	C10B—Si1B—C9B	110.1 (4)
C12A—Si1A—C11A	110.5 (4)	C11B—Si1B—C9B	106.7 (3)
C7A—N1A—H1AA	109.5	C7B—N1B—H1BA	109.5
C7A—N1A—H1AB	109.5	C7B—N1B—H1BB	109.5
H1AA—N1A—H1AB	109.5	H1BA—N1B—H1BB	109.5
C7A—N1A—H1AC	109.5	C7B—N1B—H1BC	109.5
H1AA—N1A—H1AC	109.5	H1BA—N1B—H1BC	109.5
H1AB—N1A—H1AC	109.5	H1BB—N1B—H1BC	109.5
C2A—C1A—C6A	117.7 (5)	C6B—C1B—C2B	118.5 (7)
C2A—C1A—C8A	121.1 (4)	C6B—C1B—C8B	119.7 (6)
C6A—C1A—C8A	121.2 (5)	C2B—C1B—C8B	121.8 (6)
C3A—C2A—C1A	120.3 (6)	C1B—C2B—C3B	119.6 (8)
C3A—C2A—H2AA	119.8	C1B—C2B—H2BA	120.2
C1A—C2A—H2AA	119.8	C3B—C2B—H2BA	120.2
C4A—C3A—C2A	121.3 (6)	C4B—C3B—C2B	120.6 (9)
C4A—C3A—H3AA	119.3	C4B—C3B—H3BA	119.7
C2A—C3A—H3AA	119.3	C2B—C3B—H3BA	119.7
C3A—C4A—C5A	119.1 (7)	C3B—C4B—C5B	120.4 (9)
C3A—C4A—H4AA	120.4	C3B—C4B—H4BA	119.8
C5A—C4A—H4AA	120.4	C5B—C4B—H4BA	119.8
C4A—C5A—C6A	120.5 (6)	C4B—C5B—C6B	121.1 (8)
C4A—C5A—H5AA	119.8	C4B—C5B—H5BA	119.5
C6A—C5A—H5AA	119.8	C6B—C5B—H5BA	119.5
C5A—C6A—C1A	121.0 (6)	C1B—C6B—C5B	119.7 (8)
C5A—C6A—H6AA	119.5	C1B—C6B—H6BA	120.1
C1A—C6A—H6AA	119.5	C5B—C6B—H6BA	120.1
N1A—C7A—C8A	112.8 (4)	N1B—C7B—C8B	112.0 (5)
N1A—C7A—H7AA	109.0	N1B—C7B—H7BA	109.2
C8A—C7A—H7AA	109.0	C8B—C7B—H7BA	109.2
N1A—C7A—H7AB	109.0	N1B—C7B—H7BB	109.2
C8A—C7A—H7AB	109.0	C8B—C7B—H7BB	109.2
H7AA—C7A—H7AB	107.8	H7BA—C7B—H7BB	107.9
C1A—C8A—C7A	108.5 (4)	C9B—C8B—C1B	114.1 (5)
C1A—C8A—C9A	112.4 (4)	C9B—C8B—C7B	115.0 (5)
C7A—C8A—C9A	114.2 (4)	C1B—C8B—C7B	109.1 (5)
C1A—C8A—H8AA	107.1	C9B—C8B—H8BA	105.9
C7A—C8A—H8AA	107.1	C1B—C8B—H8BA	105.9
C9A—C8A—H8AA	107.1	C7B—C8B—H8BA	105.9
C8A—C9A—Si1A	117.2 (4)	C8B—C9B—Si1B	116.8 (4)
C8A—C9A—H9AA	108.0	C8B—C9B—H9BA	108.1
Si1A—C9A—H9AA	108.0	Si1B—C9B—H9BA	108.1
C8A—C9A—H9AB	108.0	C8B—C9B—H9BB	108.1
Si1A—C9A—H9AB	108.0	Si1B—C9B—H9BB	108.1
H9AA—C9A—H9AB	107.2	H9BA—C9B—H9BB	107.3
Si1A—C10A—H10A	109.5	Si1B—C10B—H10D	109.5
Si1A—C10A—H10B	109.5	Si1B—C10B—H10E	109.5
H10A—C10A—H10B	109.5	H10D—C10B—H10E	109.5
Si1A—C10A—H10C	109.5	Si1B—C10B—H10F	109.5
H10A—C10A—H10C	109.5	H10D—C10B—H10F	109.5
H10B—C10A—H10C	109.5	H10E—C10B—H10F	109.5
Si1A—C11A—H11A	109.5	Si1B—C11B—H11D	109.5
Si1A—C11A—H11B	109.5	Si1B—C11B—H11E	109.5
H11A—C11A—H11B	109.5	H11D—C11B—H11E	109.5
Si1A—C11A—H11C	109.5	Si1B—C11B—H11F	109.5
H11A—C11A—H11C	109.5	H11D—C11B—H11F	109.5
H11B—C11A—H11C	109.5	H11E—C11B—H11F	109.5
Si1A—C12A—H12A	109.5	Si1B—C12B—H12D	109.5
Si1A—C12A—H12B	109.5	Si1B—C12B—H12E	109.5
H12A—C12A—H12B	109.5	H12D—C12B—H12E	109.5
Si1A—C12A—H12C	109.5	Si1B—C12B—H12F	109.5
H12A—C12A—H12C	109.5	H12D—C12B—H12F	109.5
H12B—C12A—H12C	109.5	H12E—C12B—H12F	109.5
			
C6A—C1A—C2A—C3A	−1.1 (10)	C6B—C1B—C2B—C3B	−2.1 (11)
C8A—C1A—C2A—C3A	179.6 (6)	C8B—C1B—C2B—C3B	176.7 (7)
C1A—C2A—C3A—C4A	−1.2 (12)	C1B—C2B—C3B—C4B	1.0 (13)
C2A—C3A—C4A—C5A	2.2 (14)	C2B—C3B—C4B—C5B	0.6 (15)
C3A—C4A—C5A—C6A	−0.7 (13)	C3B—C4B—C5B—C6B	−1.1 (15)
C4A—C5A—C6A—C1A	−1.6 (12)	C2B—C1B—C6B—C5B	1.7 (11)
C2A—C1A—C6A—C5A	2.5 (10)	C8B—C1B—C6B—C5B	−177.2 (7)
C8A—C1A—C6A—C5A	−178.2 (6)	C4B—C5B—C6B—C1B	−0.1 (13)
C2A—C1A—C8A—C7A	113.5 (6)	C6B—C1B—C8B—C9B	123.7 (7)
C6A—C1A—C8A—C7A	−65.8 (7)	C2B—C1B—C8B—C9B	−55.1 (9)
C2A—C1A—C8A—C9A	−119.2 (6)	C6B—C1B—C8B—C7B	−106.0 (7)
C6A—C1A—C8A—C9A	61.6 (7)	C2B—C1B—C8B—C7B	75.2 (7)
N1A—C7A—C8A—C1A	−175.4 (4)	N1B—C7B—C8B—C9B	−51.3 (7)
N1A—C7A—C8A—C9A	58.3 (6)	N1B—C7B—C8B—C1B	179.0 (4)
C1A—C8A—C9A—Si1A	58.3 (5)	C1B—C8B—C9B—Si1B	−59.5 (7)
C7A—C8A—C9A—Si1A	−177.4 (3)	C7B—C8B—C9B—Si1B	173.2 (4)
C10A—Si1A—C9A—C8A	163.8 (4)	C12B—Si1B—C9B—C8B	70.1 (6)
C12A—Si1A—C9A—C8A	−76.8 (4)	C10B—Si1B—C9B—C8B	−52.3 (7)
C11A—Si1A—C9A—C8A	44.9 (5)	C11B—Si1B—C9B—C8B	−171.1 (6)

### Hydrogen-bond geometry (Å, °)

**Table:**

*D*—H···*A*	*D*—H	H···*A*	*D*···*A*	*D*—H···*A*
N1A—H1AA···Cl2^i^	0.89	2.23	3.114 (5)	173.
N1A—H1AB···Cl1	0.89	2.25	3.136 (4)	172.
N1A—H1AC···Cl1^ii^	0.89	2.36	3.168 (5)	152.
N1B—H1BA···Cl2^ii^	0.89	2.30	3.166 (4)	163.
N1B—H1BB···Cl2	0.89	2.28	3.165 (4)	171.
N1B—H1BC···Cl1	0.89	2.35	3.222 (5)	165.

Symmetry codes: (i) *x*, *y*, *z*+1; (ii) *x*, −*y*+3/2, *z*+1/2.
